# Supplementary material for: Machine learning-based real-time object locator/evaluator for cryo-EM data collection
Source: Commun Biol. 2021 Sep 7;4:1044. doi: 10.1038/s42003-021-02577-1 (PMC8423793; doi:10.1038/s42003-021-02577-1)
Supplement: Supplementary file 2 — Supplementary Information [file 42003_2021_2577_MOESM2_ESM.pdf]

Supplementary Information

Machine learning-based real-time object locator / evaluator for cryo-EM data collection

Koji Yonekura [1, 2, 3, *], Saori Maki-Yonekura [1], Hisashi Naitow [1], Tasuku Hamaguchi [1], Kiyofumi Takaba [1],

[1] Biostructural Mechanism Laboratory, RIKEN SPring-8 Center, 1-1-1 Kouto, Sayo, Hyogo 679-5148, Japan

[2] Institute of Multidisciplinary Research for Advanced Materials, Tohoku University, 2-1-1 Katahira, Aoba-ku, Sendai 980-8577, Japan

[3] Advanced Electron Microscope Development Unit, RIKEN-JEOL Collaboration Center, RIKEN Baton Zone Program, 1-1-1 Kouto, Sayo, Hyogo 679-5148, Japan

* To whom correspondence should be addressed.

E-mail: yone@spring8.or.jp

Supplementary methods

Installation.

1. Download yoneoLocr-main.zip from https://github.com/YonekuraLab/yoneoLocr.
2. Extract the zip file and put the whole directory as yoneoLocr in C:\ProgramData\ of a camera control Windows PC.
3. Set the property of batch files to "full control" from the Security tab if needed.
4. Install CUDA Toolkit 10.1 and cuDNN 10.1 for a K3 control PC if the operating system of the PC is Windows Server 2012R2. Newer versions of CUDA and cuDNN are available for Windows 10.
5. Install Microsoft Build Tools for Visual Studio (vs_buildtools) if needed.
6. Install ImageMagick.
7. Launch Anaconda Prompt. Make and activate an Anaconda environment as,

    conda create -n yolov5 python=3.8

    conda activate yolov5

8. Go to the yoneoLocr directory and install python libraries as,

    conda install -c pytorch torchvision cudatoolkit=10.1

    pip install -r requirements.txt

    All required libraries are written in requirements.txt.

9. Put shortcuts, yoneoHole, yoneoXtal, yoneoDiff, and yoneoLowmagXtal on the desktop.
10. Launch yoneoLocrWatch.py from the shortcuts.

Command line options

--object hole / xtal / diff / lowmagxtal

Select running mode.

--conf-sel 0.4

A confidence threshold for object selection in hole and lowmagxtal modes. Default 0.4.

--delout yes / no

Delete output file showing objects enclosed with boxes. Default: no.

--ice yes / no

Include ice crystals for positioning in xtal mode. Default: no.

--equalize yes / no

Equalize image histogram. Only effective in hole mode. Default: yes.

Other options in the original script detect.py in YOLOv5 are also available.

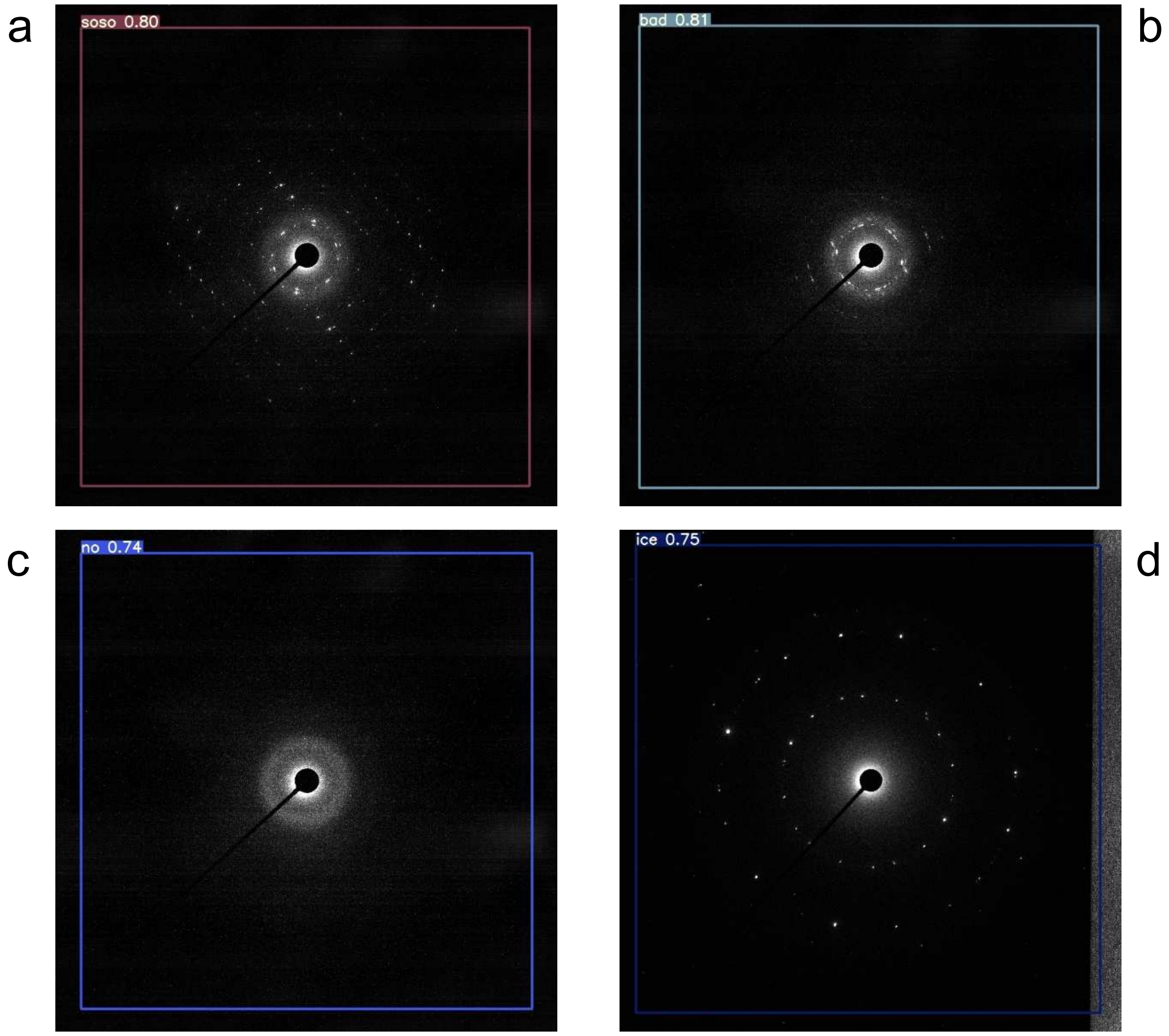

Supplementary Fig. 1. Typical evaluation results of diffraction patterns.

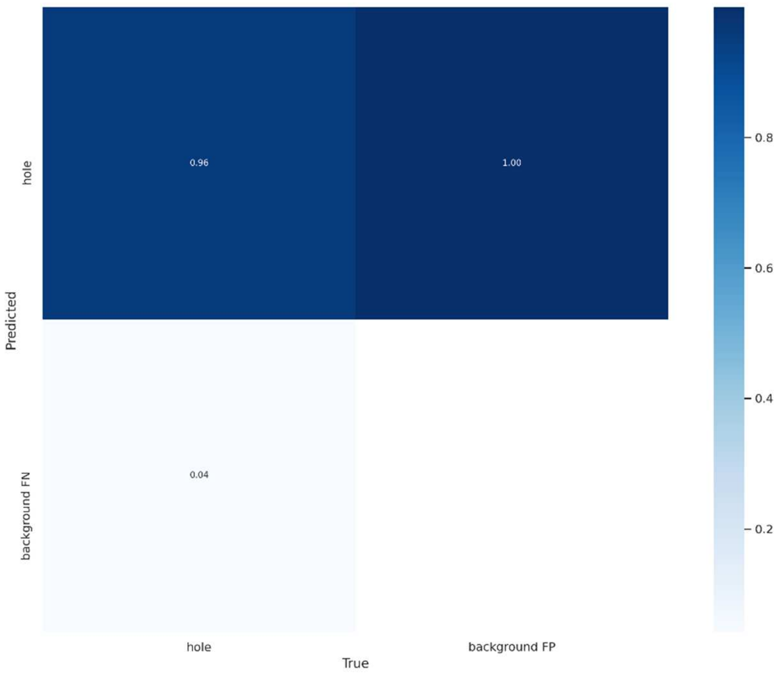

confusion_maxtrix.png for hole mode

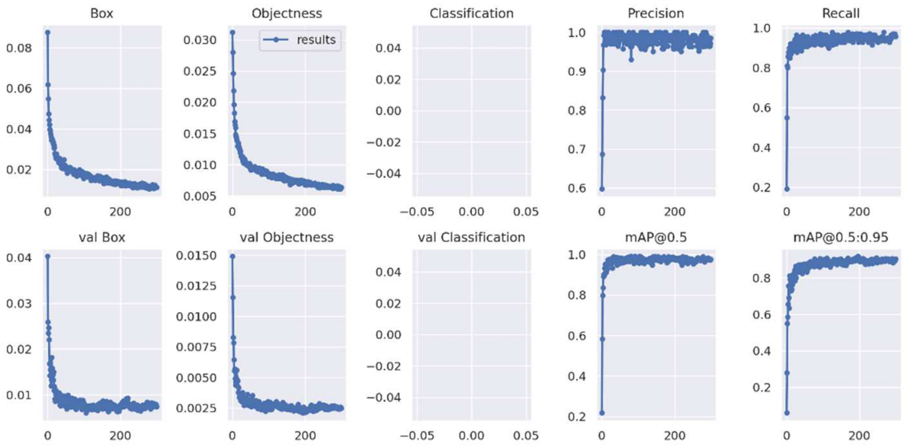

results.png for hole mode

Supplementary Fig. 2. Training metrics with YOLOv5.

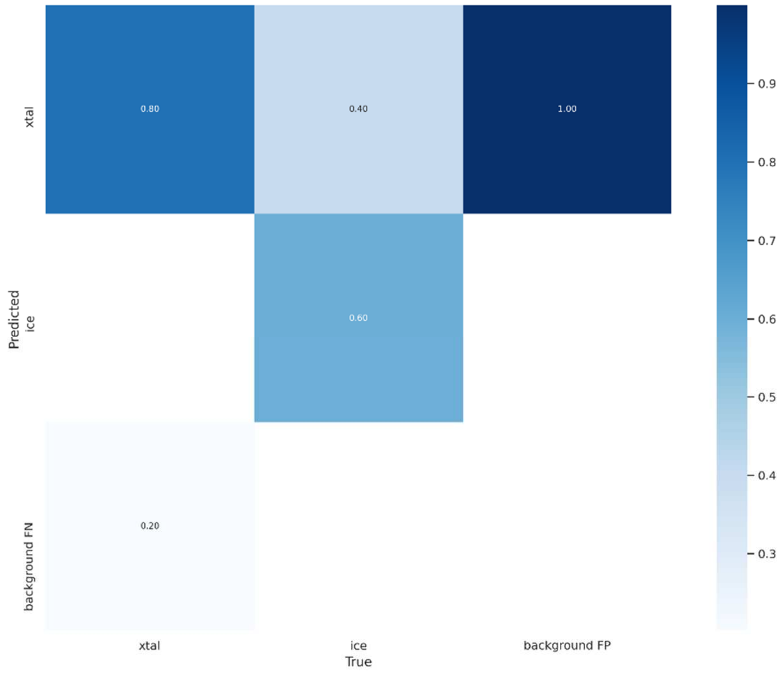

confusion_maxtrix.png for xtal mode

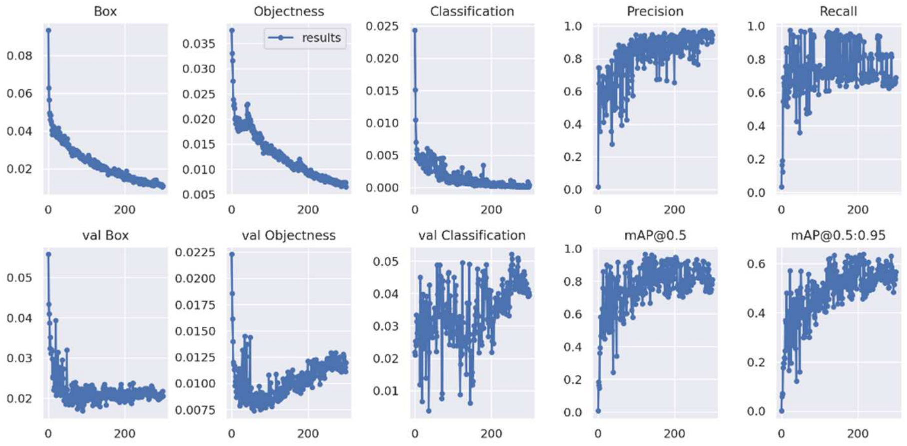

results.png for xtal mode

Supplementary Fig. 2. Continued.

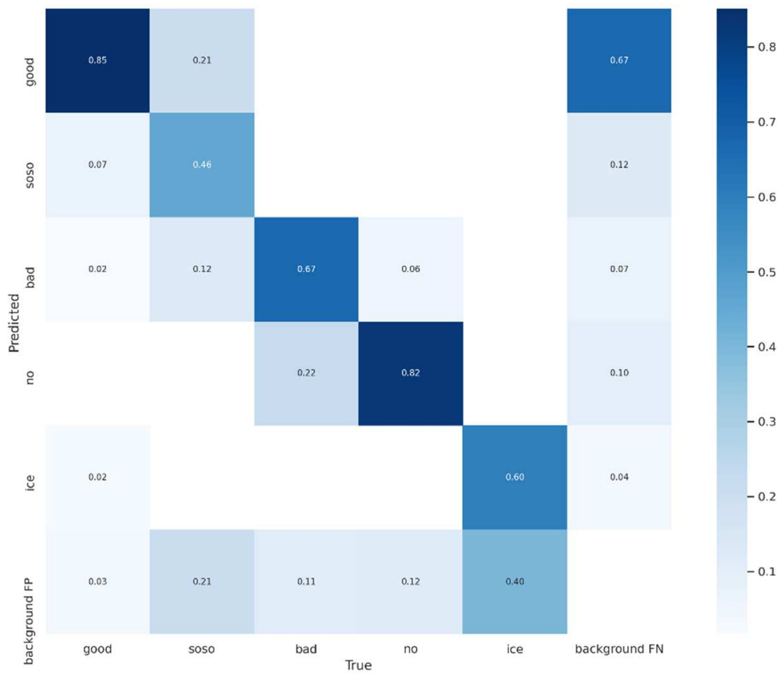

confusion_maxtrix.png for diff mode

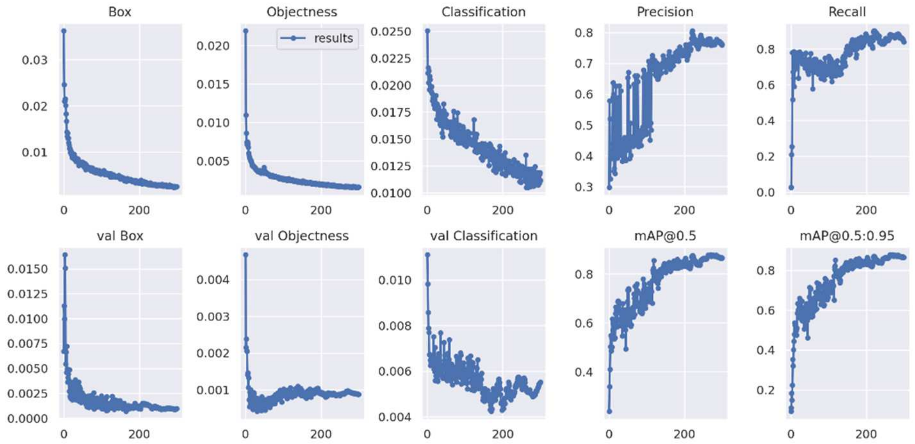

results.png for diff mode

Supplementary Fig. 2. Continued.

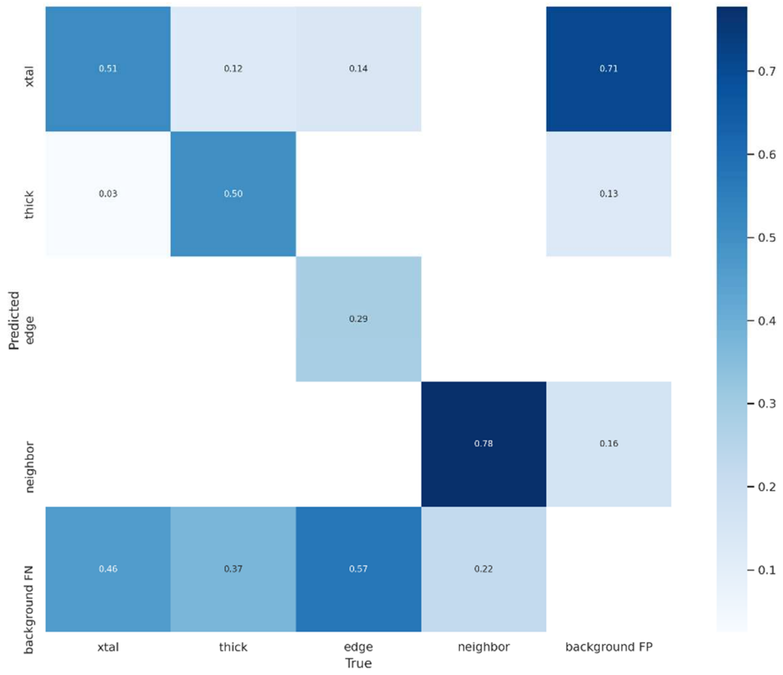

confusion_maxtrix.png for lowmagxtal mode

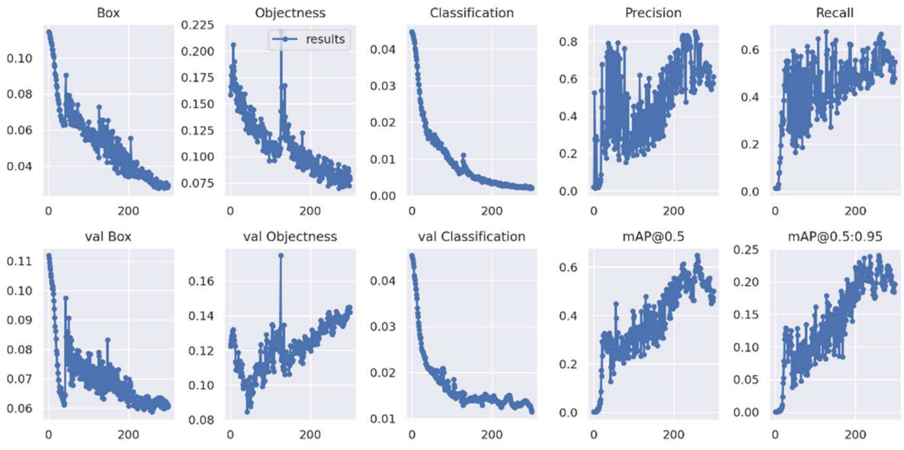

results.png for lowmagxtal mode

Supplementary Fig. 2. Continued.

Supplementary Video 1. A typical motion for hole detection and stage alignment.

Supplementary Video 2. A typical motion for crystal detection, stage alignment and diffraction evaluation.

Supplementary Video 3. A typical motion for finding crystals in low magnification images and registration of crystal positions.
