# Supplementary material for: Machine learning-based real-time object locator/evaluator for cryo-EM data collection
Source: Commun Biol. 2021 Sep 7;4:1044. doi: 10.1038/s42003-021-02577-1 (PMC8423793; doi:10.1038/s42003-021-02577-1)
Supplement: Supplementary file 3 — Description of Supplementary Files [file 42003_2021_2577_MOESM3_ESM.pdf]

Description of Additional Supplementary Files

File name: Supplementary Movie 1

Description: A typical motion for hole detection and stage alignment.

File name: Supplementary Movie 2

Description: A typical motion for crystal detection, stage alignment and diffraction evaluation.

File name: Supplementary Movie 3

Description: A typical motion for finding crystals in low magnification images and registration of crystal positions.
